# Functional Recovery of Denervated Skeletal Muscle with Sensory or Mixed Nerve Protection: A Pilot Study

**DOI:** 10.1371/journal.pone.0079746

**Published:** 2013-11-07

**Authors:** Qing Tian Li, Pei Xun Zhang, Xiao Feng Yin, Na Han, Yu Hui Kou, Jiu Xu Deng, Bao Guo Jiang

**Affiliations:** Department of Trauma and Orthopeadics, Peking University People's Hospital, Beijing, China; University of Sydney, Australia

## Abstract

Functional recovery is usually poor following peripheral nerve injury when reinnervation is delayed. Early innervation by sensory nerve has been indicated to prevent atrophy of the denervated muscle. It is hypothesized that early protection with sensory axons is adequate to improve functional recovery of skeletal muscle following prolonged denervation of mixed nerve injury. In this study, four groups of rats received surgical denervation of the tibial nerve. The proximal and distal stumps of the tibial nerve were ligated in all animals except for those in the immediate repair group. The experimental groups underwent denervation with nerve protection of peroneal nerve (mixed protection) or sural nerve (sensory protection). The experimental and unprotected groups had a stage II surgery in which the trimmed proximal and distal tibial nerve stumps were sutured together. After 3 months of recovery, electrophysiological, histological and morphometric parameters were assessed. It was detected that the significant muscle atrophy and a good preserved structure of the muscle were observed in the unprotected and protective experimental groups, respectively. Significantly fewer numbers of regenerated myelinated axons were observed in the sensory-protected group. Enhanced recovery in the mixed protection group was indicated by the results of the muscle contraction force tests, regenerated myelinated fiber, and the results of the histological analysis. Our results suggest that early axons protection by mixed nerve may complement sensory axons which are required for promoting functional recovery of the denervated muscle natively innervated by mixed nerve.

## Introduction

Peripheral nerve injury usually results in poor functional recovery, especially when nerve repair is delayed or injury is at proximal point[1].The suboptimal outcome can be attributed to degeneration of the distal nerve stump and atrophy of chronically denervated muscle [2,3]. When repair surgery is delayed, recovery is often incomplete, along with weakness and wasting [4].The capacity of motoneurons to regenerate axons into the distal segments is compromised due to prolonged denervation [5]. The distal nerve stump and skeletal muscles gradually atrophy and lose receptivity to the regenerating axon beyond a period of several months [6]. Even if the regenerating axons reach the neuromuscular junction, the function cannot be restored because of the inability of these targets to accept reinnervation [7]. With regard to the proximal injury, the recovery is likely poor although the microsurgical repair is performed at the early time. Reinnervation is necessarily prolonged as injured neurons have to regenerate their axons over a long distance. Many regenerated axons fail to reach the denervated targets for months and even years in spite of immediate repair [8].

Therefore, reinnervation within a limited time following injury is essential for maximal functional recovery. One solution, the so-called babysitter technique, is to provide the denervated segments with temporary trophic support from a foreign donor nerve until the native axons regenerate to reach the target [9]. Since this technique can protect the structure of the denervated segments, the nerve transfer repair as an alternative option may have better recovery even though the natively regenerating axons lose the capability of continuous regeneration to reinnervate target muscles in a long period. It was reported that the sensory nerve protection during chronic denervation significantly promoted skeletal muscle morphology [10,11]. However, some studies addressed that sensory babysitter technique might induce a change in Schwann cells properties in a mixed nerve stump which impairs motor axon regeneration and myelination. Sulaiman et al. observed that the reduced motor regenerated axons and remyelination of regenerated nerve fibers occurred in the presence of sensory nerve [12]. Furthermore, it has been demonstrated that the early presence of a mixed nerve improved the regenerated fibers parameter of the sciatic nerve, as well as the functional outcome in rats [13]. Therefore, the knowledge about the early presence of sensory axons leading a negative effect on functional recovery of mixed nerve repair is limited. Few studies have compared the quality of muscle recovery function in chronic denervation of mixed nerve with sensory and mixed nerve protection.

The goal of this study was to investigate whether sensory nerve protection is sufficient to improve functional recovery of target muscles following delayed denervation of mixed nerve injury. A modified babysitter technique of reverse end-to-side neurorrhaphy was used in the present study. The effects of this technique with sensory and mixed nerve protection on regenerated axons and functional recovery of the denervated targets were evaluated and compared.

## Materials and Methods

### Animals

Twenty-four adult female Sprague-Dawley rats, weighing approximately 200-250 g, obtained from the Laboratory Animal Centre of Peking University (Beijing, China) were used in this study. This study was carried out in strict accordance with recommendations in the Institutional Animal Care Guidelines and approved ethically by the Administration Committee of Experimental Animals, Peking University People’s Hospital, Beijing, China (Permit Number: 2011-16). All surgeries were performed while using a sodium pentobarbital anesthesia, and all efforts were made to minimize suffering.

### Surgical Procedures

Rats were randomly divided into four groups of six animals each. Surgeries were performed on the right hind limb of each animal.

The animals were anesthetized by an intraperitoneal injection of 2.5 % sodium pentobarbital solution (30 mg/kg body weight). All surgical procedures were performed under an aseptic manner and employed standard microsurgical techniques. The right sciatic nerve was exposed through a longitudinal incision, posterior and parallel to the femur. The nerve was dissected distal to its trifurcation to free the tibial, peroneal and sural nerves. The tibial nerve was transected 6.5 mm proximal from the entry point of the nerve into the gastrocnemius muscle. Except for the immediate repair group, the proximal stumps of the tibial nerves from the rats of the other three groups were doubly ligated and sewn into a blind-ending silicone cap to prevent regenerating fibers from reaching the distal stumps. The capped proximal stumps were then sutured on to the superfacial surface of the biceps femoris muscle.

**Figure 1:**
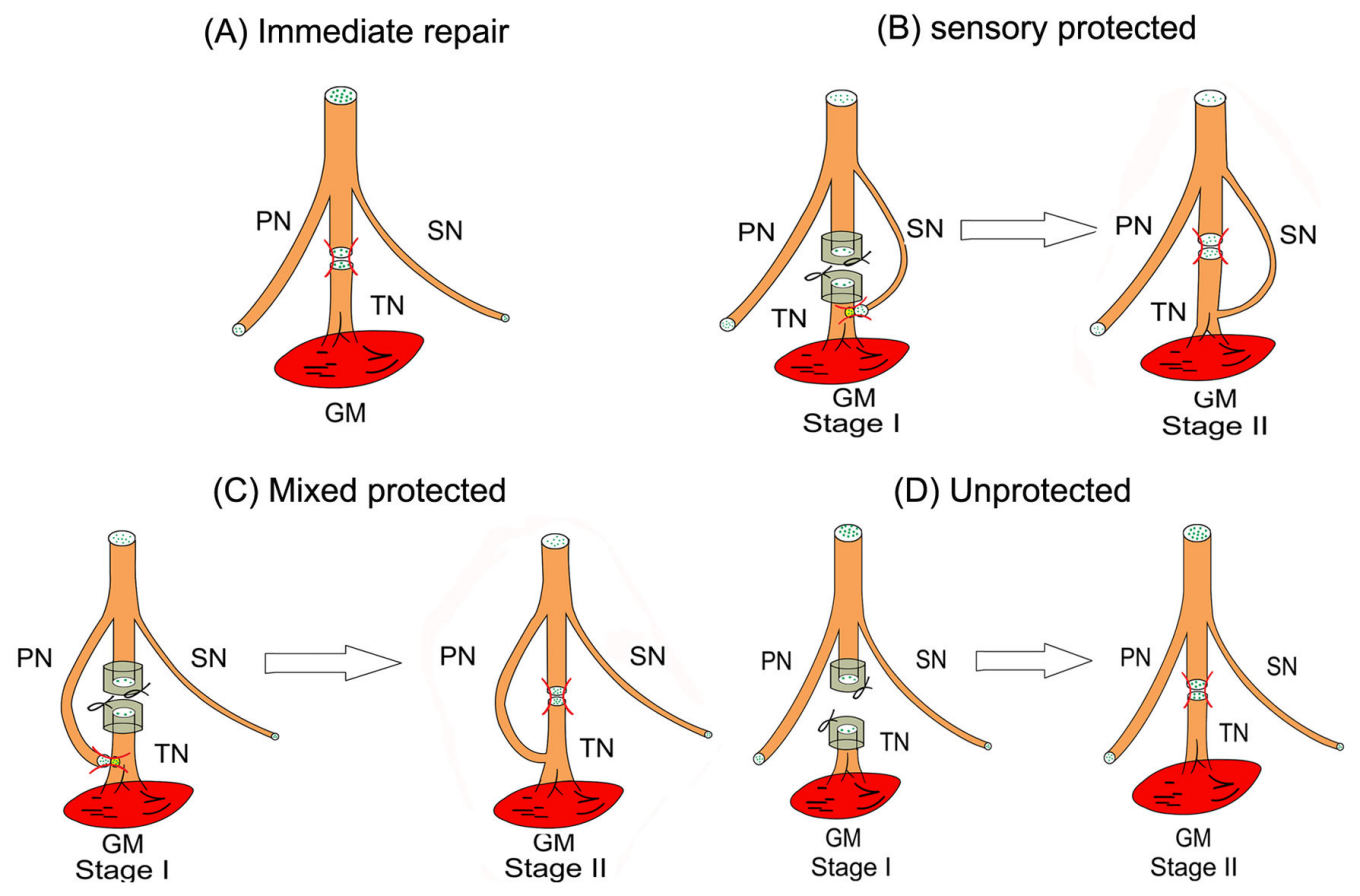
Schematic diagrams of surgical procedures producing chronic denervation of gastrocnemius muscle in the right hind limb of rats. (A) Group 1- immediate tibial nerve repair. (B) Group 2- delayed tibial nerve repair with sensory nerve protection from the sural nerve in reverse end-to-side neurorrhaphy. (C) Group 3- delayed tibial nerve repair with mixed nerve protection from the peroneal nerve in reverse end-to-side neurorrhaphy. (D) Group 4- delayed tibial nerve repair without nerve protection. (TN: tibial nerve; PN: peroneal nerve; SN: sural nerve; GM: gastrocnemius muscle).

Afterwards, four groups of the subjects received the following procedures correspondingly.. Group 1 served as positive control (immediate repair) without delayed denervation. In these animals, the epineurial sheath of the proximal stump was attached to the distal stump of the tibial nerve in an end-to-end fashion with a 10-0 nylon suture. Three months later, the rats in group 1 were sacrificed and examined (Figure 1A). For group 2 and group3, an epineurial window was created on the distal side of the tibial nerve at a site 3.5 mm proximal from the entry point of the tibial nerve into the gastrocnemius muscle. Animals in group 2 served as the experimental group of sensory nerve protection (sensoryprotection). The sural nerve was transected distally and coapted to this epineurial window in a reverse end-to-side fashion using a 10-0 nylon suture (Figure 1B). Animals in group 3 served as the experimental group of mixed nerve protection (mixedprotection). Their peroneal nerves were divided and the proximal ends were sutured reverse end-to-side to this epineurial window (Figure 1C). The distal nerve stumps in both groups were covered with a silicone cap to prevent spontaneous reinnervation by the tibial nerve. Group 4 served as the unprotected control. The muscles innervated by the tibial nerve were left denervated (Figure 1D). The surgical wound was irrigated thoroughly and closed in layers, and the animals were allowed to recover. 

Three months after the initial surgery, animals in groups 2, 3 and 4 were anesthetized again for stage II surgical procedure. Following anesthetization, the scar of distal tibial stump and the neuroma of proximal tibial stump were trimmed. Then the two stumps were sutured together in end to end neurorrhaphy (Figure 1B, C and D). The animals were allowed to recover until the testing phase. 

### Electrophysiological and Muscle Force Tests

The tibial nerves of group 1 and the other three groups on the surgery sides were exposed at three months after the first surgery and right after the second surgeries, respectively. The electrical stimuli (5V in intensity, 0.1 msec in duration, 1 Hz in frequency) (MedlecSynergy; Oxford Instrument Inc, United Kingdom) was applied to the tibial nerve trunk at positions distal and proximal to the repair site sequentially and on the donor nerve trunk 20 mm proximal to the end-to-side coaptation site. The latency and amplitude of Compound Muscle Action Potentials (CMAP) were recorded on the gastrocnemius belly at the ipsilateral side. The distance between the distal and proximal stimulation sites was measured to calculate the motor nerve conduction velocity (NCV).

Recovery of the muscle strength was determined by measuring twitch tension and tetanic tensions in the gastrocnemius muscle. For this, the muscle was freed from surrounding tissue, leaving intact only the proximal point of attachment. The knee and foot were fixed with clamps. The distal tendon of the gastrocnemius muscle was connected to force transducers (MLT500/D; Force Transducer, AD Instruments) using a nylon ligature. Hooked stimulating electrodes were placed on the tibial nerve trunk at position proximal to the repair site. A single maximal stimulus was then delivered to the tibial nerve, and the twitch tension of the entire (medial and lateral) gastrocnemius muscle was recorded at the optimal muscle length. Tetanic tension was subsequently determined with a 50-Hz electronic stimulation. The monitoring data were recorded and analyzed using the Scope software (version 3.6.12).

### Evaluation of the Gastrocnemius Muscle

Following the electrophysiological tests, the gastrocnemius muscle of the operated side was harvested and wet weights was measured. The tissue was obtain from the midbelly region and fixed in a buffered 4% paraformaldehyde solution. The tissue sample was divided into two parts. One part was washed in water, dehydrated in a graded ethanol series, cleared in xylene, embedded in paraffin and cut into 5 μm transverse sections. After the H&E staining, the sections were photographed with a DFC 300FX color digital camera (Leica, Heidelberg, Germany) to allow measurement of the cross-sectional area of muscle fiber. Images were taken of four random fields (Leica Q550 IW; Leica Imaging Systems Ltd., Cambridge, England) in four sections from every specimen, and analyzed with Leica QWin software (Leica Imaging Systems Ltd.). The second sample was flash frozen in isopentane, then cooled with solid CO_2_ and stored in a sealed plastic tube at −80°C. A serious of five longitudinal 50μm sections for each sample were cut on a freezing microtome (Frigocut, Germany) at −20°C and collected in PBS. Sections were rinsed twice in PBS, followed by 0.4% Triton-X 100 in PBS, and then incubated in 1% bovine serum albumin (BSA) and 0.05% NaN_3_ in PBS for 1 hr. The postsynapticacetylcholine receptor (AChR) site was identified by staining rhodamine-conjugated α-bungarotoxin (1:50, Molecular Probes, Eugene, OR) for 4–6 hr at room temperature. Sections were washed for 1–2 hr in several changes of PBS and mounted with Vectashield (Vector). The samples were imaged using a DMR fluorescent microscope (Leica, Wetzlar, Germany), with an excitation wavelength of 488 nm. The areas of postsynaptic receptors apposing motor endplates were digitally measured using SigmaScan Pro 5 (Systat Software Inc., San Jose, CA).

### Histological Analysis of Nerve Regeneration

As the gastrocnemius muscles were dissected, segments of the tibial nerve 2 mm distal and 2 mm proximal to the end-to-side coaptation site near its entry point into the muscle were obtained. The segments were fixed for 24 hours in 1% osmium tetroxide, dehydrated with ethanol and embedded in paraffin. The paraffin blocks were cut into 2 μm transverse sections using an ultramicrotome. Five sections from each nerve segment were randomly selected for analysis. Images were obtained using a DFC 300FX color digital camera (Leica, Heidelberg, Germany). The number of myelinated fiber, diameters of the myelinated axons and myelinated nerve fibers, and the thickness of myelin sheaths were determined from the digitized images. 

### Statistical Analysis

The results data were expressed as means ± standard deviations. The SPSS 16.0 software (SPSS Inc., Chicago, IL, USA) was employed in the data analysis. A One-Way ANOVA was utilized to analyze the experimental data. Post hoc Scheffe or Student-Newman-Keuls tests were used if further evaluations were needed. Values of P < 0.05 were considered statistically significant.

## Results

### General Observations

None of rats had systemic or regional inflammation or other post-surgical complications following the surgeries. Although the locomotor function of the operated limb was restored gradually, recovery was greater for rats which had undergone nerve protection (both sensory and mixed protection groups). 

### Electrophysiological and Muscle Force Tests

**Figure 2:**
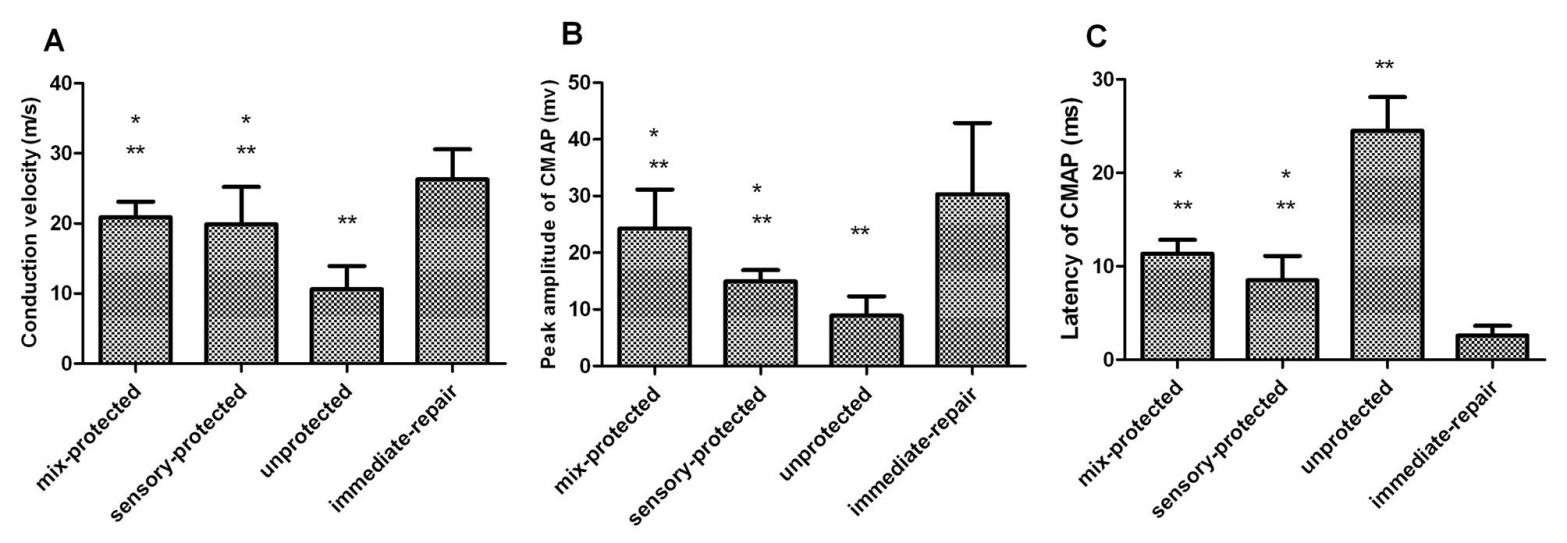
Results of electrophysiological examinations in the gastrocnemius muscle following denervation and subsequent repair with different manners. The CMAP peak amplitudes, motor nerve conduction velocities and proximal latencies among the four groups were compared. There were significant differences between the unprotected group and all other groups and between immediate-repair group and all other groups, but there were no differences between mixed-protected group and sensory-protected group (*p < 0.05 compared with unprotected control; **p < 0.05 compared with immediate-repair control).

The results of CMAP were indicated in Figure 2. In Figure 2A, the motor nerve conduction velocities of rats in the unprotected group were significantly slower than in nerve protection groups. The P value between these groups was less than 0.05. However, there was no significant difference in the conduction velocity between the two protected groups. The amplitudes can be examined both at the proximal tibial nerve (Figure 2B) and peroneal nerve (4.72 ± 0.83 mv) in mixed-protected rats. Figure 2B showed that the peak amplitude of subject in mixed nerve protection group reached 24.27 ± 6.86 mv, which was two times greater than that of the unprotected group (P<0.05). The difference in peak CMAP amplitude was not statistically significant between the mixed-protected group and sensory-protected group (Figure 2B). The proximal latencies of CMAP in three delayed repair groups significantly extended than in the immediate repair group (P< 0.05) (Figure 2C). The proximal latencies of animals in the unprotected group were about as twice as long as the mixed/sensory nerve protected groups (P<0.05) (Figure 2 C). From figure 2, it is demonstrated that the recovery level of electrophysiological properties in the immediate-repair group was significantly better than in the three delayed repair groups. This is demonstrated by observing the significant differences of the conduction velocity, peak amplitude value and the proximal latency (P< 0.05). 

The measurements of contraction force, which combines twitch and titanic tensions, are shown in Table 1. The mean tensions for animals with immediate nerve repair (1.68N~4.85N) were significantly higher than those with delayed repair (0.64N~2.76N) (P< 0.05). The contractile forces in the unprotected muscles became smaller when nerve repair was delayed for three months which is suggested by the differences between the nerve-protected and unprotected muscles (P< 0.05). The mean twitch and tetanic tensions of the gastrocnemius muscles for mixed-protected legs (1.31 ± 0.26 N, 4.05 ± 1.19 N, respectively) were considerably larger than those for sensory-protected ones (1.18 ± 0.11N, 3.79 ± 1.29N, respectively).

**Table 1:** Measurements of Contraction Force on Gastrocnemius Muscle.

Measurement (N)	Mixed-protected group	Sensory-protected group	Unprotected control	Immediate-repair
Twitch tension	1.31 ± 0.26^*†#^	1.18 ± 0.11^*†^	0.64 ± 0.02	1.68 ± 0.03
Tetanic tension	4.05 ± 1.19^*†#^	3.79 ± 1.25^*†^	2.76 ± 0.29	4.85 ± 0.89

* p < 0.05 as compared with unprotected control.

^†^ p < 0.05 as compared with immediate-repair control.

^#^ p < 0.05 as compared with sensory-protected group.

### Evaluation of the Gastrocnemius Muscle

The wet weight of gastrocnemius muscle on the operated side of the experimental rats was slightly smaller than the positive control rats which was not significant (table 2). 

**Table 2:** Measurements of Wet Weight and Cross –Sectional area on Gastrocnemius Muscle.

Measurement	Mixed-protected group	Sensory-protected group	Unprotected control	Immediate-repair
Wet weight (g)	0.97 ± 0.23	0.94 ± 0.33	0.92 ± 0.35	1.01 ± 0.42
Cross-sectional area (µm^2^)	666.29 ± 247.03^*^	638.63 ± 369.32^*^	449.06 ± 150.77	875.71 ± 329.56

* p < 0.05 as compared with unprotected control.

**Figure 3:**
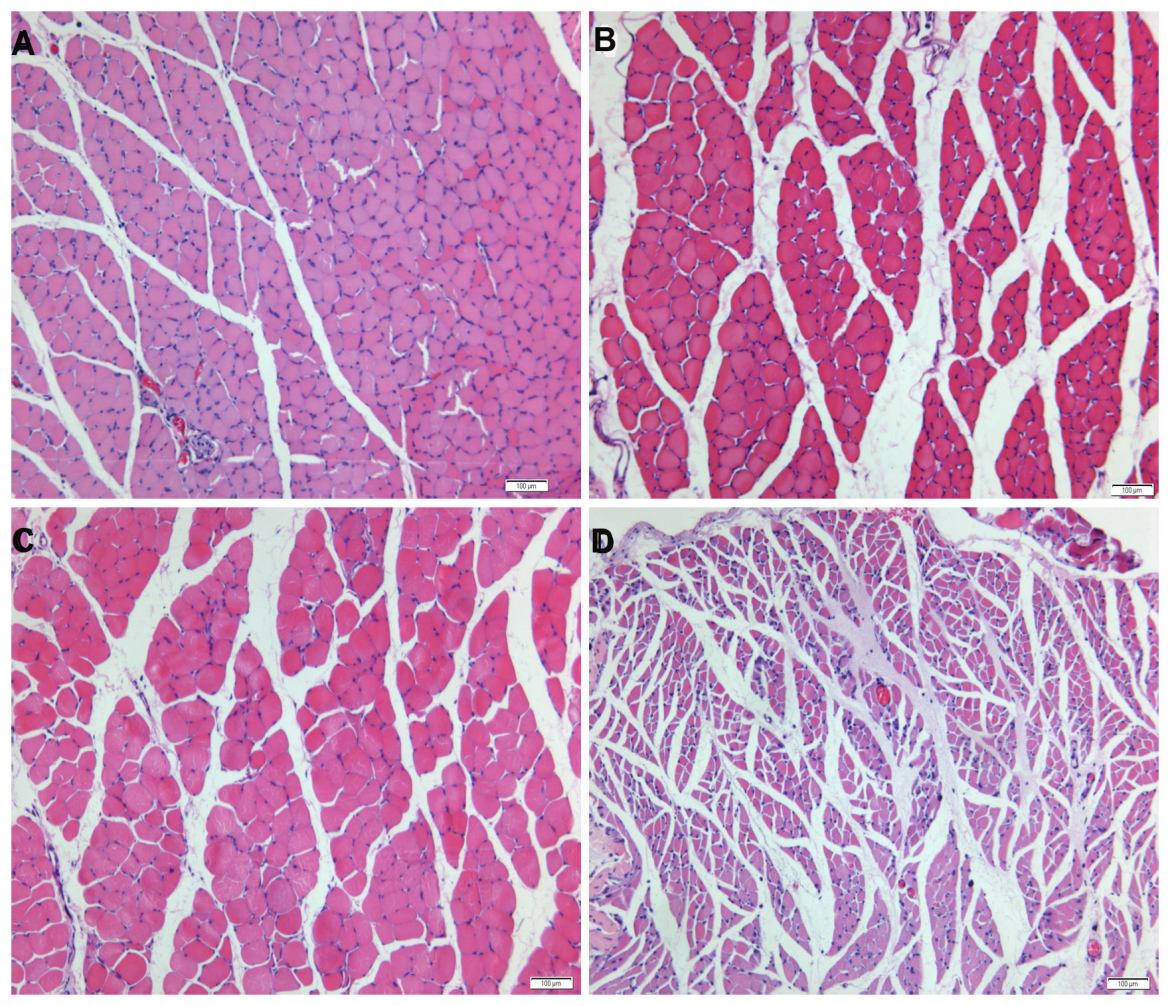
Photomicrographs of transverse sections of the gastrocnemius muscle following denervation and subsequent reinnervation. The muscle sections of (A) immediate repair, (B) chronic denervation with sensory protection and (C) chronic denervation with mixed-nerve protection showed wide areas of larger muscle fibers among small groups of atrophied fibers. (D) Unprotected muscle sections showed extensive atrophy. Magnification×100. Scale bar= 100µm.

The transverse sections of the gastrocnemius muscle for the four groups’ subjects were displayed in Figure 3. In the immediate repair group, the muscle fibers and surrounding connective tissue were indistinguishable from normal tissue (Figure 3A). The ultrastructure was also preserved in muscles with sensory-nerve and mixed-nerve protection, although a greater variation in fiber cross-sectional area and a slight increase in the amount of connective tissue were observed (Figure 3B, C). The fiber cross-sectional area in the mix-protected group and sensory-protected group was 666 ± 247 μm^2^ and 638 ± 369 μm^2^, respectively. The values in the two protected groups were not significantly different. In contrast, the muscle in the unprotected group had large regions of atrophied fibers and connective tissue hyperplasia (Figure 3D). The cross-sectional area of muscle fibers was significantly smaller in the unprotected group than in the nerve-protected groups (table 2).

**Figure 4:**
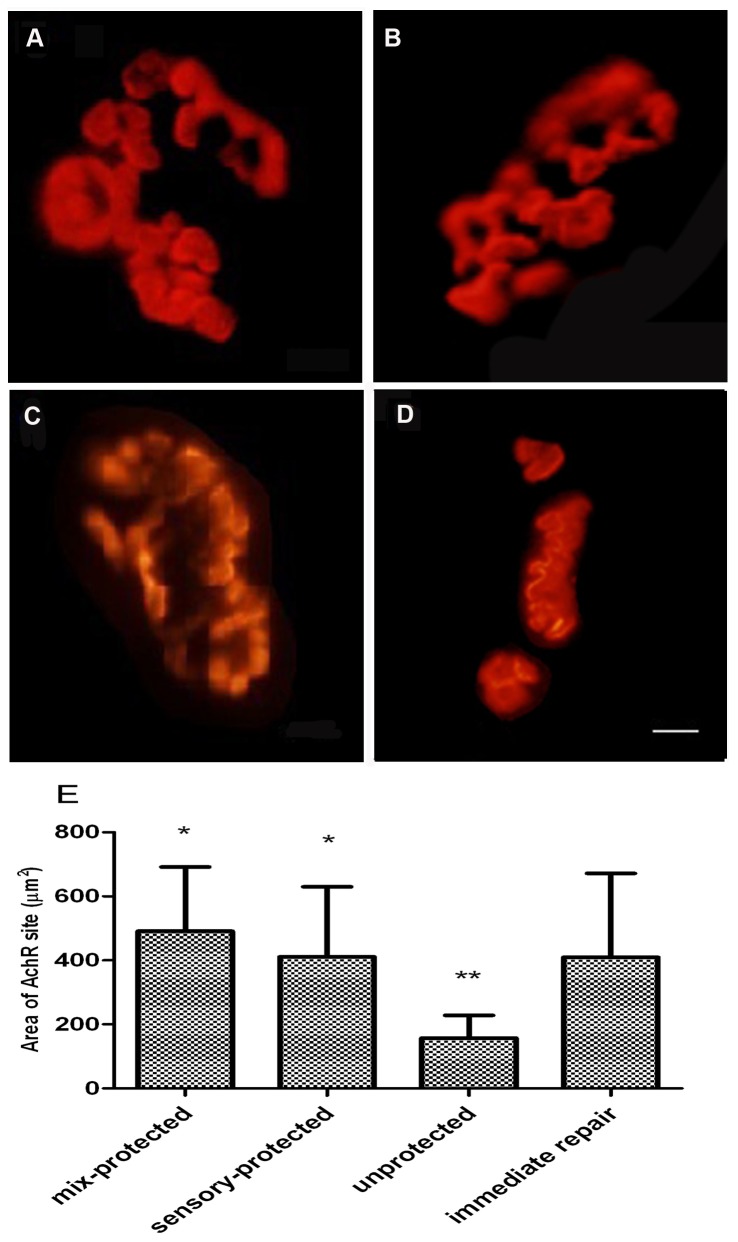
Postsynaptic AChR plaques at motor endplates in gastrocnemius muscle, as visualized by α-bungarotoxin binding. (A) AChR plaques in the immediate repair group had well-defined contours. The appearance of AChR plaques in (B) chronic denervation with sensory protection and (C) chronic denervation with mixed-nerve protection displayed thick fringes and a few small round cupulae with distinct contours. The appearance of AChR plaques in (D) the unprotected group appeared as delineated, small, flat and slender. Magnification×400. Scale bar= 5µm. (E) Graphs showed that the area of AChR sites in the unprotected group was significantly smaller than in the other groups. (*p < 0.05 compared with unprotected control; **p < 0.05 compared with immediate-repair control).

In the evaluation of motor endplates, abnormal morphology at postsynaptic AChR sites was observed (Figure 4). A majority of the AChR plaques in the unprotected group appeared to be less delineated, small, flat and slender, while displaying the appearance of shrinkage and hypersegmentation. Disorganization, reduced density, and fragmentation of the AChR plaque into abundant patches were also observed (Figure 4D). The area of AChR sites was smaller in the unprotected group than in the nerve-protected groups. In contrast, the AChR plaques in protected groups were well-defined, similar to those in the immediate repair group (Figure 4A), displaying thick fringes and a few small round cupulae with distinct contours (Figure 4 B, C). The difference in the areas of the AChR site for the two protected groups was not statistically significant. 

### Histological Analysis for Nerve Regeneration

**Figure 5:**
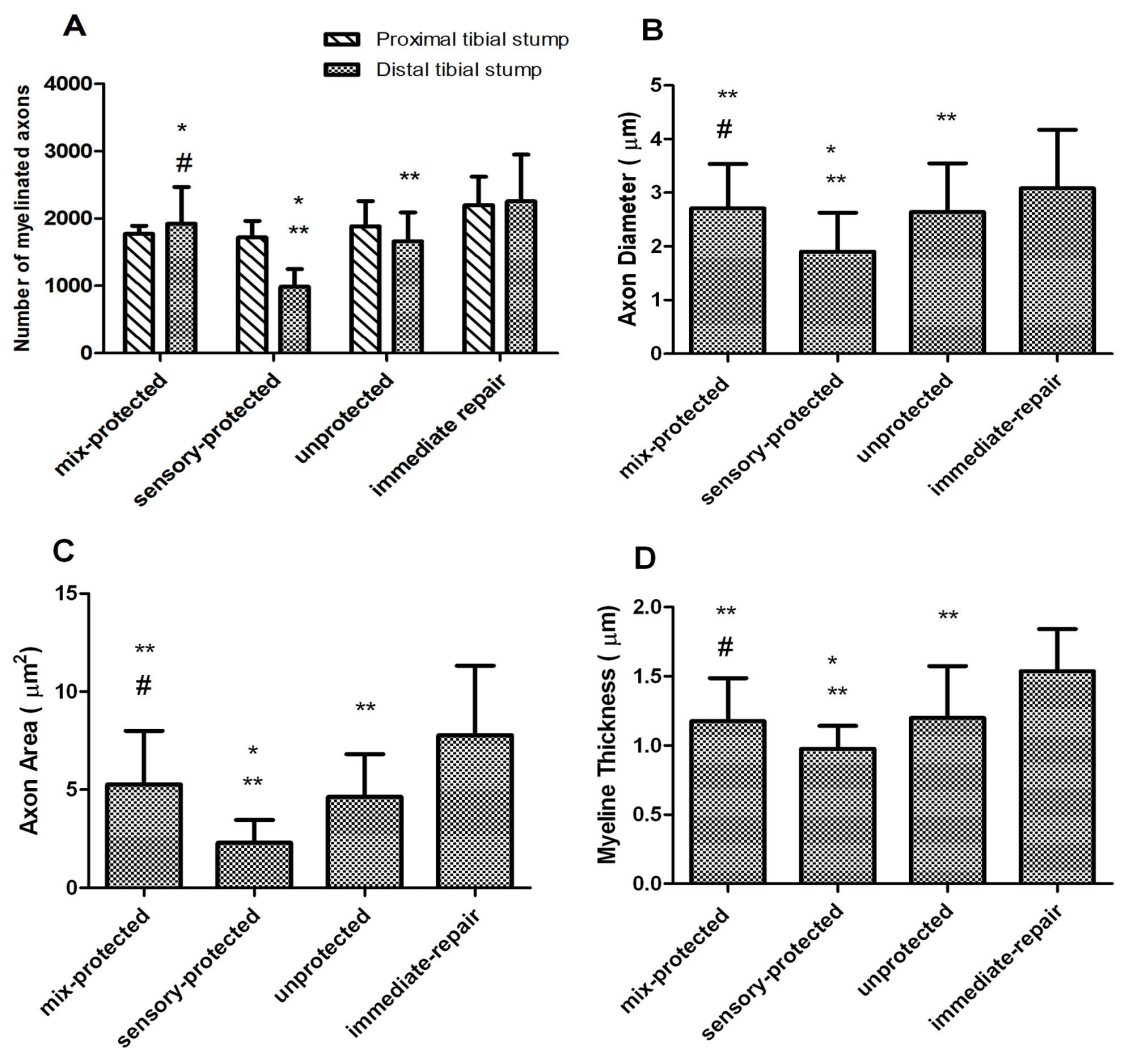
Histograms depicting the assessment of the regenerated tibial nerve parameters. (A) The number of myelinated axons were not significantly different among the four groups at the proximal region of the tibial nerve. At the distal transaction, the immediate-repair and mixed-protected rats had significantly greater number of regenerated myelinated axons than the unprotected ones. There were significantly fewer numbers of regenerated myelinated axons in the sensory-protected group. (B) The axonal diameter and (C) area of myelinated fibers were smaller and (D) the width of myelin sheath was thinner in sensory-protected group (*p < 0.05 compared with unprotected control; **p < 0.05 compared with immediate-repair control; # p < 0.05 compared with sensory-protected group).

Osmium tetroxide staining indicated that the number of myelinated axons at the proximal region of the tibial nerve was similar for the four groups (Figure 5A). 

**Figure 6:**
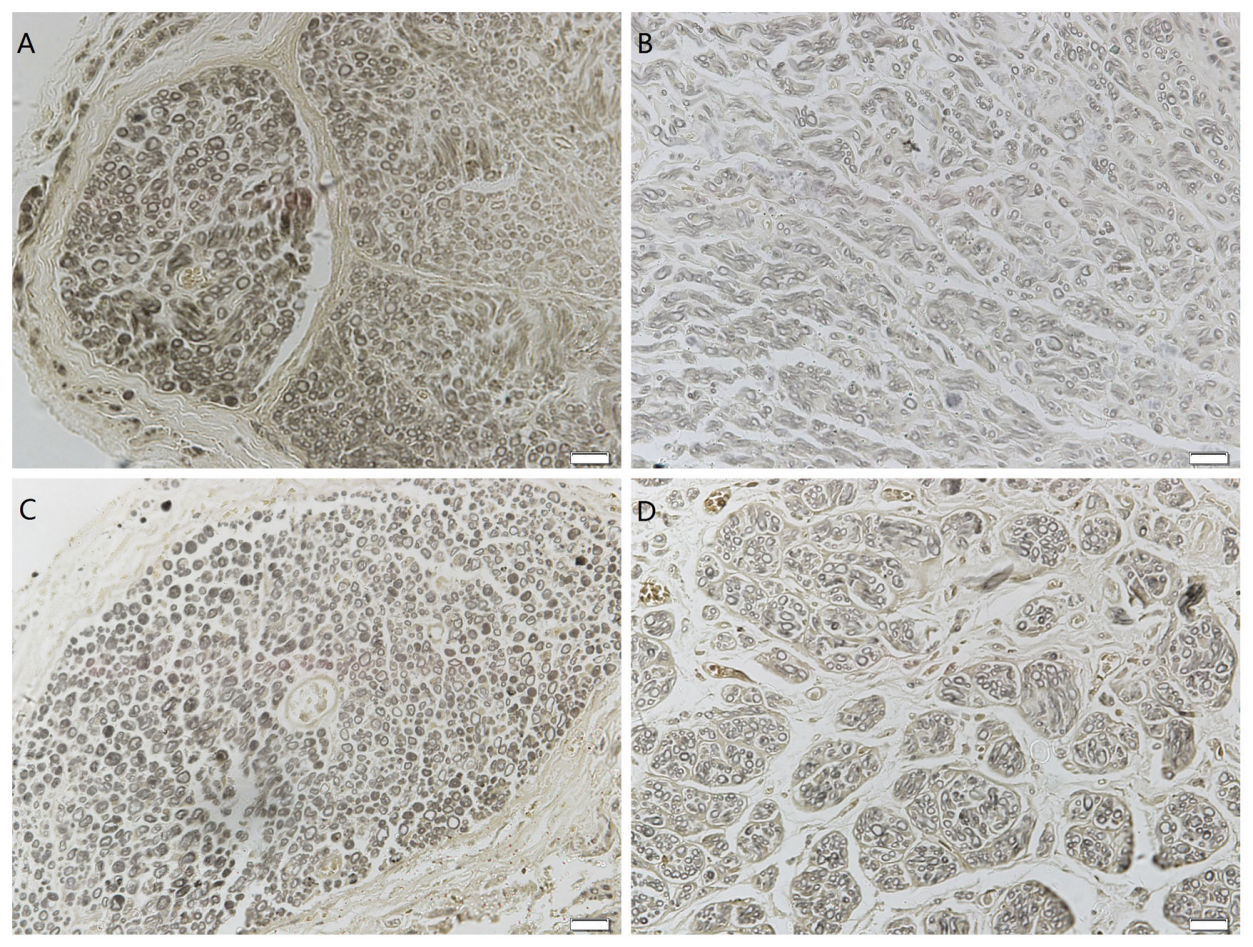
Photomicrographs of the distal portion of the regenerated tibial nerve. (A) Immediate repair showed good myelin regeneration. The diameters and thicknesses of the myelin sheathes were uniform and regular. (B) Chronic denervation with sensory protection displayed poor myelin regeneration, with an uneven distribution and a low density. A low level of myelin degeneration and necrosis was observed. The diameters and thicknesses in the sensory protection group were uneven and generally smaller than in the group of (C) chronic denervation with mixed-nerve protection. (D) The myelin distribution of delayed repair without protection was irregular. The diameters and thicknesses of the myelin sheathes in the unprotected group were generally smaller than in the mixed-protected group, and were larger than in the sensory-protected group. Magnification×400. Scale bar=20µm.

The tibial stump distal to the suture site in each group revealed a regeneration of clustered myelinated fibers (Figure 6). The immediate-repair and mixed-protected rats had significantly greater number of regenerated myelinated axons than the unprotected ones (Figure 5A), which displayed robust myelination and axonal integrity (Figure 6A, C). Interestingly, significantly fewer number of regenerated myelinated axons were observed in the sensory-protected group (985 ± 264) compared to those in the mixed-protected group (1923 ± 544) (Figure 5A). Light microscopic examination showed significant evidence of myelin and axonal degeneration in the sensory-protected rats (Figure 6B).The axonal diameter (Figure 5B) and area of myelinated fibers (Figure 5C) were smaller and the width of myelin sheath (Figure 5D) was thinner in the sensory-protected group than in the mixed-protected group.

## Discussion

Peripheral nerve injuries present a complex reconstructive challenge when reinnervation is delayed, either due to the timing of the surgical intervention or the long distance that must be traveled by the regenerating nerve fiber [1]. Proximal nerve injuries always result into poor outcomes because of the insufficient time to innervate motor endplates. With a low rate of axon regeneration [5], there is a limited chance of significant recovery when nerve regeneration occurs over a long distance [8]. The restoration of muscle function negatively correlates with the duration of denervation [2]. Typically, motor endplates lose the capacity to reinnervate target sites after several months. To overcome this problem, the axon–endplate connections should be reestablished as soon as possible following injury. The so-call baby-sitter technique, which entails early innervation by a foreign donor nerve until a native axon can regenerate to reach the target, reportedly prevents atrophy of the denervated muscles [9–11]. In the present study, we compared the quality of functional recovery in chronically denervated segments with sensory or mixed nerve protection following tibial nerve injury in rats. 

The present data revealed that muscle fiber architecture was preserved by early innervation from either sensory or mixed nerve. Both early innervation of sensory and mixed nerve had better functional recovery after reinnervation by native axons. Interestingly, repair with mixed nerve protection was significantly superior to repair with sensory nerve protection in greater number of regenerated myelinated axons, larger axonal diameter and myelinated fibers area, thicker myelin sheath, as well as greater functional recovery of muscle fibers.

The conventional babysitter technique makes temporary use of a foreign donor nerve to neurotize the distal nerve stump in end-to-end neurorrhaphy. When regenerated axons from the native proximal nerve stump reach the target muscle, the donor nerve is cut and cross-sutured with the native nerve stump [5,9,10]. In this method, distal nerve stumps twice undergo Wallerian degeneration prior to target reinnervation by native axons, adversely affecting axonal regeneration and functional recovery. Yoshimura et al. [14] reported that two sequential episodes of skeletal muscle denervation and reinnervation led to greater force and power deficits than a single peripheral nerve injury and repair in rats. An alternative procedure in the current study was used to connect the donor nerve to the distal nerve stump in a reverse end-to-side neurorrhaphy. In this method, denervated muscles are protected until axons of the native nerve reach their target without the necessity for a second operation [15]. The present findings in electrophysiological tests demonstrated that both tibial and peroneal axons had achieved noteworthy reinnervation of the target muscle. Thus, the reverse end-to-side neurorrhaphy is an effective technique for early innervation of the target muscle without impeding native axons regeneration [16].

Previous studies found deterioration of trophic and substrate support for regenerating axons in chronically denervated targets. The absence of trophic and substrate support from the distal stumps, and the atrophy of the muscle together undermine the reinnervation of a muscle from regenerated axons of native motoneurons [17,18]. Following acute nerve injury, Schwann cells in the distal nerve stumps undergo proliferation and phenotypic changes to prepare a local environment favorable to axonal regeneration. However, these changes are transient and gradually decrease with an increase in the duration of denervation [19,20]. Over time, chronically denervated muscles become less receptive to regenerated motor axons because of a significant loss of viable muscle cells resulting from a combination of fiber necrosis, connective tissue hyperplasia, and exhaustion of satellite cells [4,11,21]. The loss of input from neurotransmitters, neurotrophic factors, and other signals, also contributes to muscle fiber atrophy and diminish receptivity to regenerated axons [11]. The deterioration of the motor endplate also undermines functional recovery following prolonged denervation [22]. In the present experiments, unprotected muscles had large numbers of atrophied fibers and connective tissue hyperplasia, which is consistent with previous studies [9–11]. In contrast, with sensory-nerve or mixed-nerve protection the structure and cross-sectional area of the target muscle, as well as the areas postsynaptic to the motor endplate were preserved. Although the exact mechanism by which early innervation by foreign nerves preserves the capacity for muscle reinnervation is not fully understood, it may maintain endoneurial pathways and prolong the growth-supportive phenotype of Schwann cells to allow timely reinnervation [9]. Donor axons may directly or indirectly support denervated muscle fibers by the supply of trophic factors, improve existing endoneurial sheath structure and enhance regeneration by the native nerve [11,23]. Timely reinnervation induces Schwann cell proliferation [24] and their expression of neurotrophins such as NGF, BDNF, GDNF, and pleotrophin as well as their receptors, and also neuregulin and the cognate receptors [25,26]. All of these factors, which are associated with neuronal survival and axonal growth in the peripheral nerves system, may promote the reestablishment of a functional connection at the neuromuscular junction [27–29].

In addition of the preservative effect of the muscle fiber architecture, our findings suggested that early innervation with sensory or mixed nerve had a better functional recovery following subsequent native nerve reinnervation. The results of a benefit with early innervation were in agreement with recent peer studies [9–11]. The viable muscle fibers were small after long-term denervation and its reversal in size relied on incorporation of satellite cells which were considered as the main source of additional nuclei to growing fibers in postnatal muscles [30–32]. However, the satellite cell pool is likely exhausted in prolonged denervation [33,34]. Furthermore, the present data revealed that repair with mixed nerve protection was significantly superior to repair with sensory nerve protection in terms of twitch and tetanic tensions. Although a number of studies demonstrated the protected effect of babysitter technique on denervated muscle fiber structure, few studies reported the quality of functional recovery with different types of nerves protection. In this study, a positive effect of mixed nerve protection on functional restoration of mixed nerve injury was observed. The exact mechanism of these findings is unknown. A few studies have shown that early presence of sensory axons may induce a change in Schwann cell properties in a mixed nerve stump which impairs motor axon regeneration and myelination. In this study, the rats of mixed-nerve protection group had remarkably regenerated myelinated axons, larger axonal diameter and an area of myelinated fiber, and thicker myelin sheath than the sensory-nerve protection group. Neurotization by a mixed nerve prior to native axons reinnervation may be more effective in promoting reinnervation than neurotization by sensory nerve. Schwann cells express distinct sensory or motor characteristics that support regeneration in a phenotype-specific manner [35]. The motor phenotype can be modified toward the sensory phenotype through forcible innervation by sensory axons [35]; however, sensory nerves may induce a phenotype in Schwann cells that make them less capable of regenerating motor axons [12]. This distinction between sensory and motor Schwann cells may explain the less robust reinnervation that was observed after long-term denervation in the absence of sensory axons. Thus, Schwann cells of mixed nerves seem to be more permissive to regeneration from mixed axons than those of sensory axons. This possibility need to be clarified in future studies. Although the muscle architecture protected by the sensory nerve was similar to that protected by the mixed nerve, the twitch and tetanic tensions of reinnervated muscle fibers cannot be completely reversed in sensory protection. This is possibly due to the reduced number of regenerated axons which reinnervated the target muscle fibers [4,36,37]. In summary, our results suggest that the sensory axons are not sufficient and early mixed axons protection is required to improve functional recovery of chronically denervated muscles natively innervated by the mixed nerve. 

## Conclusion

Early innervation with a donor nerve in reverse-end-to-side neurorrhaphy helped to preserve the structure of chronically denervated muscle fibers. The functional recovery of the denervated targets natively innervated by the mixed nerve was significantly promoted by the mixed nerve protection. This technique could be helpful in the setting of prolonged denervation following peripheral nerve injury, although its clinical applicability remains to be investigated. 
